# Comparison of nucleoside and nucleotide analogs in the recurrence of hepatitis B virus‐related hepatocellular carcinoma after surgical resection: A multicenter study

**DOI:** 10.1002/cam4.4348

**Published:** 2021-10-13

**Authors:** Weili Qi, Junyi Shen, Junlong Dai, Youwei Wu, Yu Zhang, Shusheng Leng, Fengwei Gao, Shun Ran, Wei Peng, Xiaoyun Zhang, Tianfu Wen, Chuan Li

**Affiliations:** ^1^ Department of Liver Surgery and Liver Transplantation Center West China Hospital of Sichuan University Chengdu Sichuan China; ^2^ Organ Transplantation Center Sichuan Provincial People’s Hospital Chengdu Sichuan China; ^3^ Department of Hepatopancreatobiliary Surgery Affiliated Hospital of Chengdu University Chengdu Sichuan China; ^4^ HBPS Diseases Center for Diagnosis and Treatment of Leshan City People’s Hospital of Leshan Leshan Sichuan China; ^5^ Department of Hepatopancreatobiliary Surgery Affiliated Cancer Hospital of Guizhou Medical University Guiyang, Guizhou China

**Keywords:** hepatitis B virus, hepatocellular carcinoma, nucleos(t)ide analogs, recurrence, resection

## Abstract

**Background:**

Antiviral therapy should reduce the recurrence of hepatitis B virus‐related hepatocellular carcinoma (HBV‐related HCC) after surgical resection. However, there is little research on whether various antiviral drugs have different prognostic effects in patients with HBV‐related HCC after curative liver resection. The present study compared the effects of nucleotide analog (NtA) and nucleoside analog (NsA) antiviral therapies after surgical resection on the prognosis of HBV‐related HCC.

**Methods:**

A total of 1303 patients with HBV‐related HCC who received curative hepatectomy at five institutes between April 2014 and April 2019 were retrospectively enrolled and analyzed. Propensity matching analysis was used to compare the outcomes of HCC patients given NsA versus NtA therapy. Subgroup analysis of patients treated with entecavir (ETV) and tenofovir disoproxil fumarate (TDF) was also performed.

**Results:**

Among 1303 patients, 759 (58.2%) patients developed recurrence, and 460 (35.3%) patients died. Multivariable analyses revealed that NtA therapy significantly decreased the risk of HCC recurrence (hazard ratio [HR], 0.64; 95% confidence interval [CI], 0.51–0.80; *p* < 0.001) and HCC‐related death (HR, 0.52; 95% CI, 0.36–0.76; *p* = 0.001) compared to that with NsA therapy. Subgroup analysis showed that TDF treatment was associated with significantly lower rates of HCC recurrence (HR, 0.64; 95% CI, 0.49–0.83; *p* = 0.001) and death (HR, 0.32; 95% CI, 0.20–0.50; *p* < 0.001) than ETV treatment.

**Conclusions:**

Nucleotide analog treatment, but not NsA treatment, significantly reduced the risk of HCC recurrence in patients with HBV‐related HCC and improved overall survival after curative hepatic resection.

## Graphical abstract

The aim of this study was to investigate the effect of antiviral drug on the prognosis of hepatitis B virus‐related hepatocellular carcinoma (HCC) after curative hepatectomy. We found that nucleotide analog treatment, rather than nucleoside analog treatment, significantly reduced HCC recurrence and improved overall survival after curative hepatic resection.

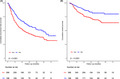

## 1 INTRODUCTION

Hepatocellular carcinoma (HCC) is the sixth most frequently diagnosed cancer and the third most frequent cause of death worldwide. HCC is the fourth most common malignant tumor in China and the second leading cause of death^1^, ^2^ largely because hepatitis B virus (HBV) infection, which is the major cause of HCC development, is epidemic in China.^3^, ^4^ Hepatectomy is the mainstay of HCC treatment, and it leads to expected outcomes (5‐year survival of 60%–80%) in well‐selected candidates.^5^, ^6^ However, a high recurrence rate of approximately 60% after curative hepatic resection at 5 years is the main factor affecting the prognosis of HCC.^7^ High serum levels of HBV‐DNA are an important predictor of HCC recurrence.^8^, ^9^ Previous well‐designed studies demonstrated that antiviral therapy reduced the risk of postoperative recurrence of HBV‐related HCC.^10^, ^11^, ^12^ Recently, Choi et al. found that patients treated with tenofovir disoproxil fumarate (TDF) had a lower risk of postoperative HCC recurrence than those treated with entecavir (ETV),^13^ but Lee et al. found no significant difference in the risk of recurrence and death between the ETV and TDF groups.^14^ Currently, direct‐acting antivirals include nucleotide analogs (NtAs) and nucleoside analogs (NsAs). The commonly used nucleos(t)ide analogs (NUCs) that act as direct‐acting antivirals include but are not limited to TDF and ETV. Additionally, the potential impact of direct‐acting antivirals on HCC recurrence is still a controversial topic. It is still unclear whether NtAs and NsAs have different effects on HCC recurrence rates in patients with HBV‐related HCC after curative resection.

Therefore, we aimed to compare the different effects of NtAs and NsAs on HCC recurrence and overall survival (OS) in patients with HBV‐related HCC after curative resection.

## 2 MATERIALS AND METHODS

### 2.1 Study population

**FIGURE 1:**
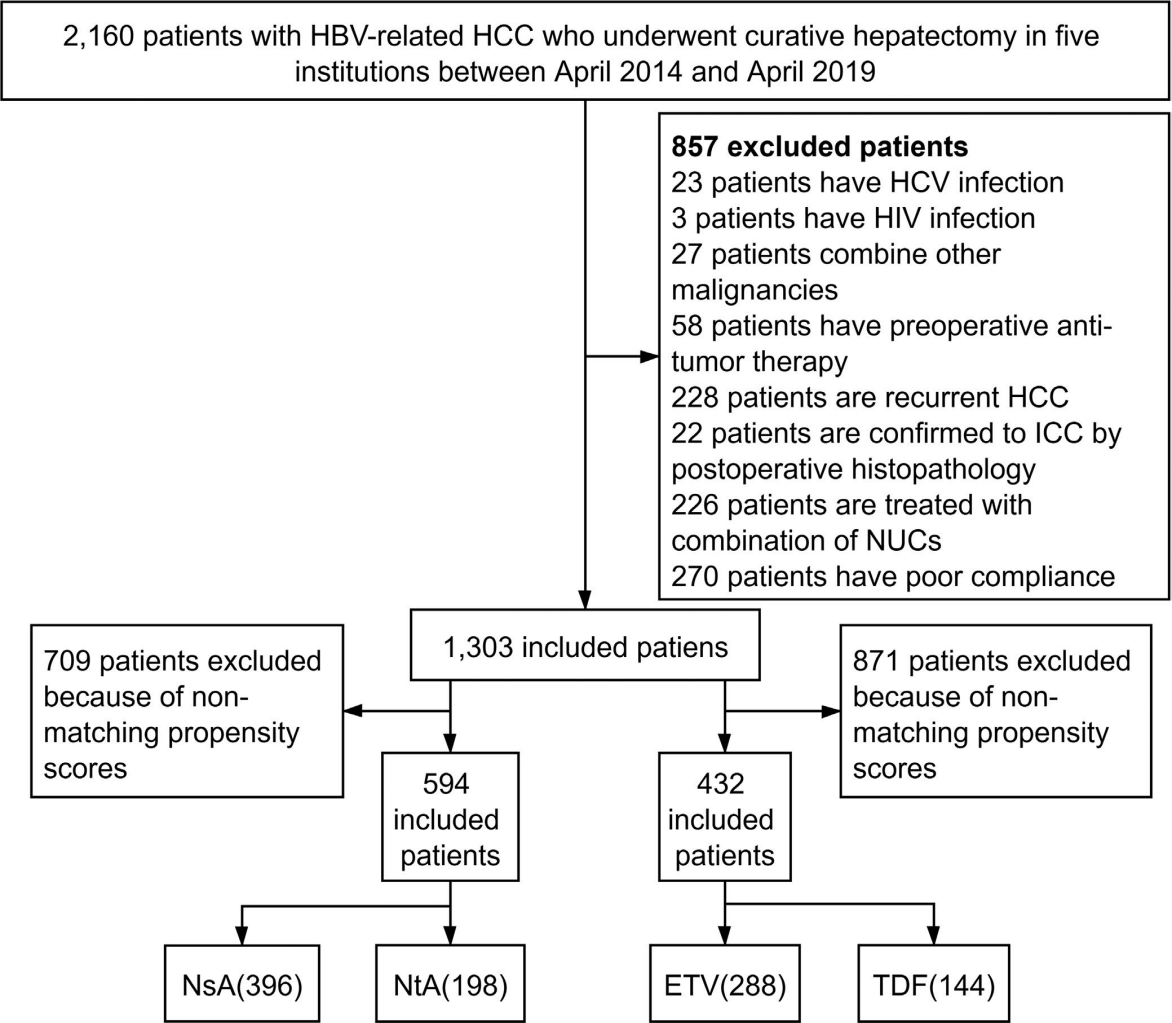
Flowchart of the process for patient selection

Using a retrospectively collected database, we identified patients with HBV‐associated HCC who underwent therapeutic liver resection in five participating institutions between April 2014 and April 2019 (Figure 1). The participating institutions included The Affiliated Cancer Hospital of Guizhou Medical University, the Affiliated Hospital of Chengdu University, the People's Hospital of Leshan, Sichuan Provincial People's Hospital, and West China Hospital of Sichuan University. HBV‐related HCC was defined as progression to HCC after the detection of serum hepatitis B surface antigen (HBsAg) for at least 6 months.^15^, ^16^ The patients were divided into NsA and NtA groups.

We excluded patients who satisfied any of the following criteria: (1) other concurrent malignancies or recurrent HCC; (2) coinfection with other viruses (e.g., hepatitis C virus, hepatitis D virus, or human immunodeficiency virus); (3) preoperative antitumor treatment; (4) use of other antiviral therapies, such as interferon; (5) treatment with a combination of NUCs; (6) no/irregular treatment with NUCs; and (7) poor liver function (Child‐Turcotte‐Pugh [CTP] class C, Figure 1).

Baseline patient and tumor characteristics were obtained from electronic medical records at each medical center and included patient demographics, HBsAg, hepatitis B virus e antigen, serum HBV‐DNA load, alpha fetoprotein (AFP), coagulation function, liver function, renal function, and hematological parameters. Hepatic pathologists assessed tumor characteristics in the excised specimens. The antiviral choice for each patient was based on their socioeconomic status and the preferences of each doctor.

This study was performed according to the World Medical Association Declaration of Helsinki and approved by the Ethics Committee on Biomedical Research, West China Hospital of Sichuan University (IRB No. 2021‐311).

### 2.2 Follow‐up

All patients were followed up at 1 month postoperatively, every 3 months for the first 3 years and every 6 months for the next few years. The tumor evaluation and follow‐up protocol included multiphasic contrast‐enhanced computed tomography (CT) or magnetic resonance imaging (MRI), physical examination, blood cell and differential counts, liver function tests, AFP levels, HBV markers, and HBV‐DNA levels.

The primary endpoint was recurrence‐free survival (RFS), and the secondary endpoint was OS. The index date was defined as the date of hepatectomy for HCC. RFS was defined as the interval between surgery and the first incidence of positive recurrence. OS was defined as the time interval between surgery and death of any cause or last follow‐up. The last date of follow‐up was 31 March 2021.

Tumor recurrence was defined as multiphasic contrast‐enhanced CT or MRI showing intrahepatic lesions with typical HCC enhancement characteristics, that is, contrast enhancement in the arterial phase and washout in the venous phase.

### 2.3 Statistical analysis

To balance the baseline characteristics and minimize the effect of potential confounders, nearest‐neighbor 1:2 propensity score matching (PSM) with a caliper size of 0.02 was used to reduce group differences in covariances between NsA and NtA patients (Figure 1). Propensity scores were calculated using the following 20 variables: age, sex, Barcelona Clinic Liver Cancer (BCLC) stage, tumor size, tumor number, microvascular invasion (MVI), diabetes, hypertension, serum HBV‐DNA level, HBsAg, AFP, prothrombin time (PT), red blood cell count, hemoglobin, white blood cell count, platelets, aspartate aminotransferase (AST), alanine aminotransferase, albumin (ALB), and total bilirubin.

Baseline characteristics were grouped into continuous and categorical variables. Continuous variables, which are reported as the means ± standard deviation, were compared between groups using the *t*‐test or the Mann–Whitney *U* test. Categorical variables were compared using the χ^2^ test or Fisher’s exact test, and the results are expressed as numbers (*n*) or proportions (%). Cumulative RFS and OS rates were analyzed using the Kaplan–Meier method, and differences were compared using the logarithmic rank test. Univariable and multivariable Cox proportional risk regression analyses were performed to identify predictors associated with RFS and OS and assess risk factors that lead to recurrence and death.

All statistical analyses were performed using R statistical software version 4.04 (R Foundation for Statistical Computing) and SPSS software version 25.0 (SPSS). A two‐tailed *p* value < 0.05 was considered statistically significant.

## 3 RESULTS

### 3.1 Patient characteristics

The study included 1303 patients: 1105 (84.8%) received NsA postoperatively; and 198 (15.2%) received NtA postoperatively. Table 1 shows the patient characteristics for the entire cohort. The median age was 51.2 years, and 1101 (84.4%) patients were male. The patients in NsA group had lower levels of ALB (*p* = 0.022), higher levels of AFP (*p* = 0.034), higher levels of AST (*p* < 0.001), longer PT (*p* = 0.0025), and larger tumor size (*p* < 0.001) compared with NtA group. More patients in the NsA group had higher serum HBV‐DNA levels (>1000 IU/ml; *p* < 0.001) and later BCLC stage (BCLC stage B and C; *p* = 0.003).

**TABLE 1:** Baseline characteristics and clinical data

Characteristics	Entire cohort (*n* = 1303)			Propensity score‐matched cohort A (198 pairs, *n* = 594)			Propensity score‐matched cohort B (144 pairs, *n* = 432)		
	NsA (*n* = 1105)	NtA (*n* = 198)	*p* value	NsA (*n* = 396)	NtA (*n* = 198)	*p* value	ETV (*n* = 288)	TDF (*n* = 144)	*p* value
Age, years	51.4 ± 11.1	50.4 ± 10.2	0.265	50.4 ± 11.6	50.4 ± 10.3	0.985	49.3 ± 10.6	49.9 ± 10.7	0.610
Male sex, *n* (%)	936 (84.7)	165 (83.3)	0.623	323 (81.5)	165 (83.3)	0.596	247 (85.7)	122 (84.7)	0.772
Hypertension, *n* (%)	312 (28.2)	45 (22.7)	0.110	99 (25.0)	45 (22.7)	0.542	57 (19.7)	28 (19.4)	0.932
Diabetes mellitus, *n* (%)	138 (12.4)	25 (12.6)	0.957	44 (11.1)	25 (12.6)	0.587	43 (14.9)	20 (13.8)	0.772
BMI, kg/m^2^	23.1 ± 3.1	23.2 ± 3.0	0.630	23.1 ± 3.1	23.2 ± 3.0	0.895	23.1 ± 3.1	23.4 ± 3.1	0.301
HBeAg positive, *n* (%)	248 (22.4)	42 (21.2)	0.701	90 (22.7)	42 (21.2)	0.675	56 (23.0)	29 (24.1)	0.806
HBV DNA, IU/ml	1.05 × 10^6^ ± 4.42 × 10^6^	7.40 × 10^5^ ± 3.98 × 10^6^	0.420	7.24 × 10^5^ ± 3.57 × 10^6^	7.40 × 10^5^ ± 3.98 × 10^6^	0.966	1.01× 10^6^ ± 3.74 × 10^6^	1.01 × 10^6^ ± 4.70 × 10^6^	0.988
HBV DNA > 10^3^ IU/ml, *n* (%)	670 (60.6)	91 (45.9)	<0.001	189 (47.7)	91 (45.9)	0.684	157 (54.5)	76 (52.7)	0.733
AFP, ng/ml	2983.58 ± 19277.00	1127.69 ± 7039.56	0.034	1899.08 ± 111221.32	1127.69 ± 7039.56	0.432	2331.38 ± 17205.12	1000.52 ± 7551.85	0.443
AFP > 400 ng/ml, *n* (%)	461 (41.7)	77 (38.8)	0.456	150 (37.8)	77 (38.8)	0.811	109 (37.8)	58 (40.2)	0.625
Total bilirubin, mg/dl	16.28 ± 17.48	14.35 ± 5.73	0.124	14.35 ± 6.19	14.35 ± 5.73	0.991	14.48 ± 6.90	14.34 ± 5.91	0.834
Albumin, g/L	42.29 ± 5.07	43.18 ± 4.65	0.022	43.33 ± 5.52	43.18 ± 4.65	0.750	43.18 ± 5.28	43.39 ± 4.49	0.675
ALT, IU/ml	50.47 ± 56.41	46.41 ± 34.93	0.327	44.60 ± 38.12	46.41 ± 34.94	0.575	49.50 ± 49.35	48.58 ± 36.75	0.843
AST, IU/ml	53.71 ± 53.41	43.45 ± 28.14	<0.001	41.43 ± 23.46	43.35 ± 28.14	0.379	46.25 ± 36.11	44.58 ± 29.55	0.632
PT, s	12.6 ± 3.1	12.3 ± 1.0	0.025	12.3 ± 2.4	12.3 ± 1.0	0.775	12.1 ± 0.9	12.2 ± 0.9	0.521
RBC, ×1000/mm^3^	4.72 ± 0.67	4.68 ± 0.61	0.352	4.68 ± 0.61	4.68 ± 0.61	0.946	4.70 ± 0.61	4.70 ± 0.60	0.994
WBC, ×1000/mm^3^	6.85 ± 22.34	5.28 ± 2.12	0.321	5.17 ± 1.74	5.28 ± 2.12	0.524	5.47 ± 1.99	5.35 ± 2.19	0.585
PLT, ×1000/mm^3^	146.6 ± 74.7	137.9 ± 70.1	0.126	137.7 ± 68.9	137.9 ± 70.1	0.973	142.6 ± 68.2	144.8 ± 71.5	0.754
Hemoglobin, g/L	143.4 ± 19.0	142.8 ± 18.8	0.707	143.8 ± 18.8	142.8 ± 18.8	0.547	143.4 ± 19.9	143.7 ± 18.6	0.850
BCLC stage									
Very early (0)	60 (5.4)	22 (11.1)		33 (8.3)	22 (11.1)		18 (6.2)	10 (6.9)	
Early (A)	824 (74.5)	148 (74.7)	0.003	300 (75.7)	148 (74.7)	0.332	212 (73.6)	107 (74.3)	0.702
Intermediate (B)	67 (6.0)	9 (4.5)		28 (7.0)	9 (4.5)		19 (6.5)	8 (5.5)	
Advanced (C)	154 (13.9)	19 (9.5)		35 (8.8)	19 (9.5)		39 (13.5)	19 (13.1)	
Single tumor, *n* (%)	1001 (90.5)	178 (89.8)	0.761	352 (88.8)	178 (89.8)	0.708	253 (87.8)	128 (88.8)	0.752
Tumor size, cm	6.5 ± 4.1	5.2 ± 3.6	<0.001	5.2 ± 3.3	5.2 ± 3.6	0.989	5.4 ± 3.3	5.6 ± 3.8	0.506
Cirrhosis, *n* (%)	932 (84.3)	173 (87.3)	0.274	349 (88.1)	173 (87.3)	0.790	243 (84.3)	120 (83.3)	0.781
MVI, *n* (%)	294 (26.6)	49 (24.7)	0.584	103 (26.0)	49 (24.7)	0.740	87 (30.2)	44 (30.5)	0.941
Capsular invasion, *n* (%)	500 (45.2)	97 (48.9)	0.331	180 (45.4)	97 (48.9)	0.416	127 (44.0)	71 (49.3)	0.306
Satellite nodules, *n* (%)	136 (12.3)	15 (7.5)	0.055	35 (8.8)	15 (7.5)	0.601	22 (7.6)	11 (7.6)	>0.999
Tumor differentiation									
Low	497 (44.9)	94 (47.4)	0.501	182 (45.9)	94 (47.4)	0.709	155 (53.8)	76 (52.7)	0.766
Intermediate	594 (53.7)	102 (51.5)		209 (52.7)	102 (51.5)		132 (45.8)	66 (45.8)	
High	14 (1.2)	2 (1.0)		5 (1.2)	2 (1.0)		1 (0.3)	2 (1.3)	

Abbreviations: AFP, alpha‐fetoprotein; ALT, alanine aminotransferase; AST, aspartate aminotransferase; BCLC, Barcelona Clinic Liver Cancer staging system; BMI, body mass index; ETV, entecavir; HBeAg, hepatitis B e antigen; MVI, microvascular invasion; NsA, nucleoside analog; NtA, nucleotide analog; PLT, platelets; PT, prothrombin time; RBC, red blood cell count; TDF, tenofovir disoproxil fumarate; WBC, white blood cell count.

Propensity score matching adjustment resulted in 594 patients, and none of the parameters of the two groups continued to be significantly different (Table 1). The characteristics between patients treated with ETV and TDF were not significantly different (Table 1).

### 3.2 Difference in RFS based on NsA or NtA therapy

**FIGURE 2:**
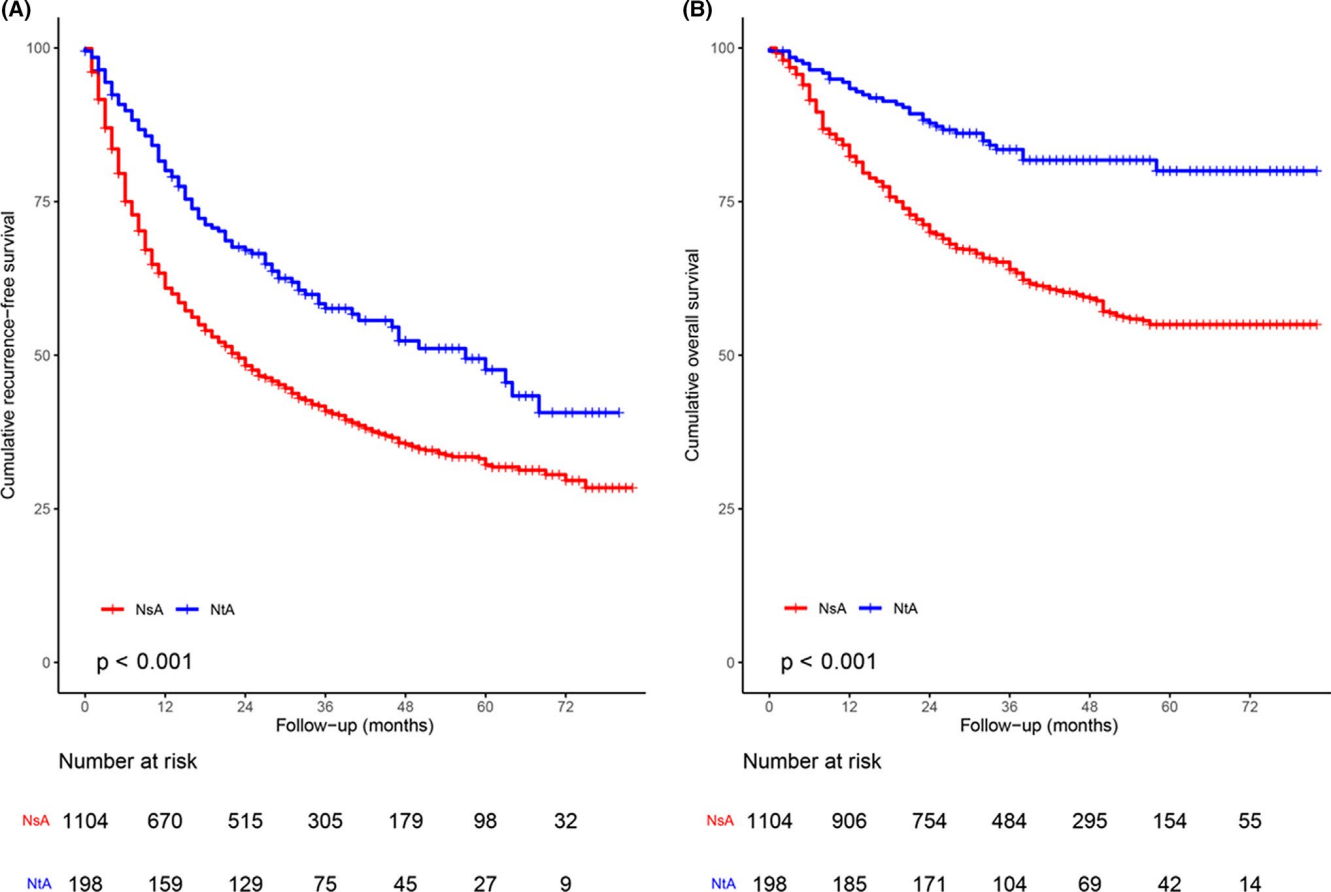
Kaplan–Meier analysis of (A) RFS and (B) OS among 1303 patients receiving NsA treatment or NtA treatment. NsA, nucleoside analog; NtA, nucleotide analog; OS, overall survival; RFS, recurrence‐free survival

With a median follow‐up time of 47.0 months for the 1303 patients, 759 (58.2%) patients developed recurrence. RFS in NtA group was significantly longer than in NsA group (hazard ratio [HR], 0.60; 95% confidence interval [CI], 0.49–0.75; *p* < 0.001; Figure 2A). The cumulative recurrence rates were 36.1%, 56.5%, and 65.5% at 1, 3, and 5 years, respectively (Figure 2A).

**FIGURE 3:**
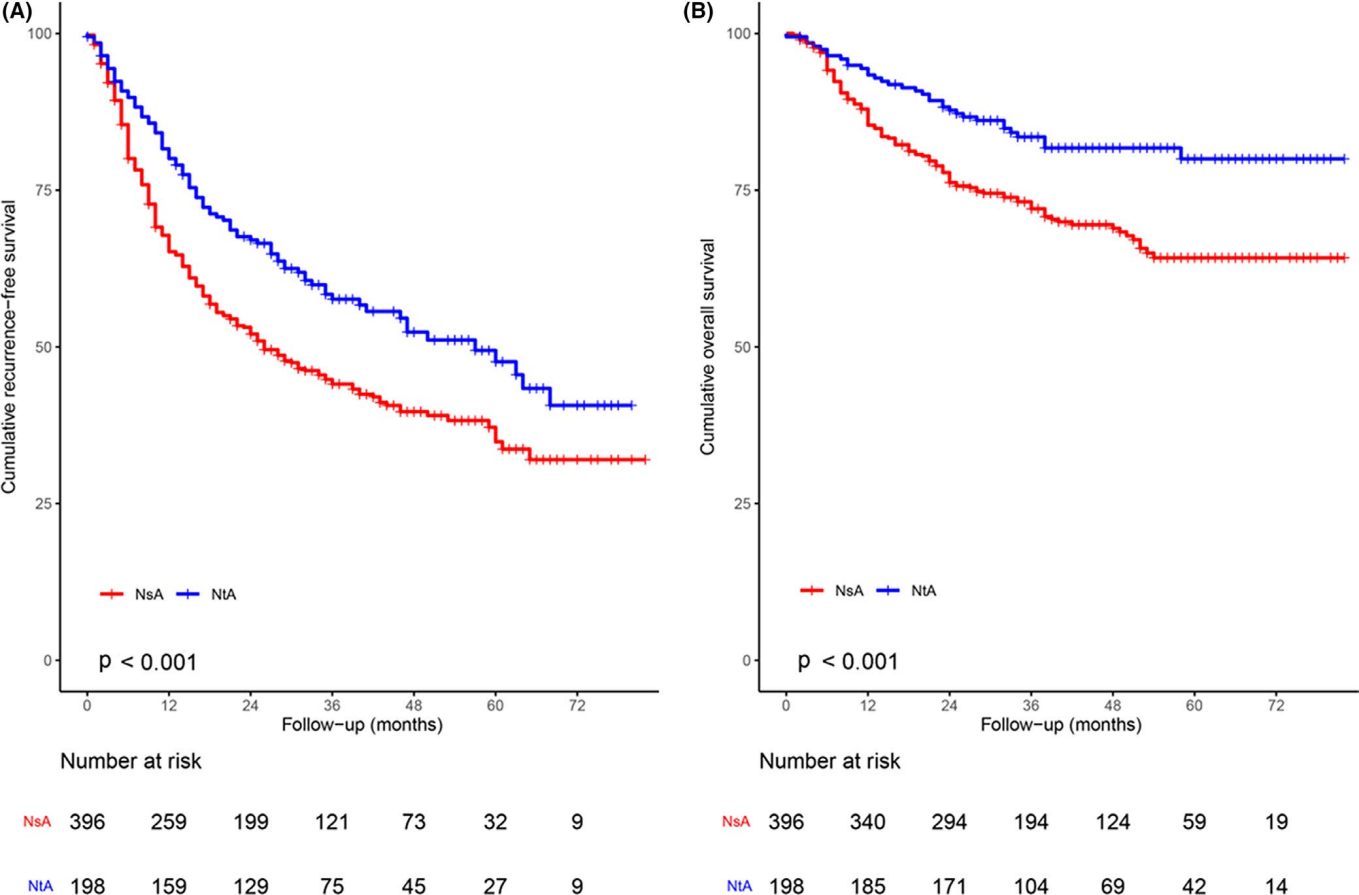
Kaplan–Meier analysis of (A) RFS and (B) OS among 594 patients receiving NsA treatment or NtA treatment. NsA, nucleoside analog; NtA, nucleotide analog; OS, overall survival; RFS, recurrence‐free survival

Of the 567 PSM patients (396 in the NsA group and 198 in the NtA group), 228 (57.5%) patients in the NsA therapy group developed recurrence, and 90 (45.4%) patients in the NtA therapy group developed recurrence. There was a significant difference in RFS between patients who received NsA and NtA therapy, and the NtA group had a better RFS rate (HR, 0.67; 95% CI, 0.52–0.85; *p* = 0.001; Figure 3A). The 1‐, 3‐, and 5‐year recurrence rates in the NsA treatment group were 34.8%, 55.9%, and 65.1%, respectively, and 19.9%, 42.4%, and 52.4%, respectively, in the NtA treatment group (*p* = 0.001, Figure 3A).

Multivariate Cox regression analyses of 1303 patients were performed to determine RFS predictors of recurrence in patients with HBV‐related HCC after hepatectomy, and parameters significantly associated with recurrence risk in univariate analysis were incorporated into multivariate analysis. The multivariate Cox regression model included the entire cohort of 1303 patients. NtA treatment was associated with a significantly lower risk of HCC recurrence than NsA treatment (HR, 0.64; 95% CI, 0.51–0.80; *p* < 0.001; Table 2), independent of other predictive factors. Independent risk factors for HCC recurrence included younger age (HR, 1.22; 95% CI, 1.02–1.48; *p* = 0.03), later stage of BCLC (HR, 1.30; 95% CI, 1.06–1.61, *p* = 0.01), larger tumor size (HR, 1.78; 95% CI, 1.51–2.08, *p* < 0.001), multiple tumors (HR, 1.33; 95% CI, 1.03–1.73; *p* = 0.03), cirrhosis (HR, 1.52; 95% CI, 1.20–1.92; *p* < 0.001), MVI (HR, 1.52; 95% CI, 1.28–1.80; *p* < 0.001), capsular invasion (HR, 1.18; 95% CI, 1.02–1.37; *p* = 0.03), presence of satellite nodules (HR, 1.30; 95% CI, 1.05–1.60; *p* = 0.02), lower preoperative ALB level (HR, 1.22; 95% CI, 1.04–1.43; *p* = 0.02), and higher preoperative AST level (HR, 1.48; 95% CI, 1.15–1.90; *p* = 0.002; Table 2).

**TABLE 2:** Multivariate analyses of RFS and OS in HCC patients receiving NsA or NtA therapy after hepatectomy

Variables	RFS		OS	
	MV HR (95% CI)	MV *p* value*	MV HR (95% CI)	MV *p* value*
Age (≤60 vs. >60 years)	1.22 (1.02–1.48)	0.03	1.23 (0.97–1.56)	0.09
BCLC stage (B, C vs. 0, A)	1.30 (1.06–1.61)	0.01	2.06 (1.61–2.62)	<0.001
Group (NtA vs. NsA)	0.64 (0.51–0.80)	<0.001	0.41 (0.29–0.59)	<0.001
Tumor size (>5.0 vs. ≤5.0 cm)	1.78 (1.51–2.08)	<0.001	1.87 (1.51–2.33)	<0.001
Multiple tumors (Yes vs. No)	1.33 (1.03–1.73)	0.03	0.87 (0.63–1.19)	0.38
Cirrhosis (Yes vs. No)	1.52 (1.20–1.92)	<0.001	1.70 (1.22–2.37)	0.002
MVI (Yes vs. No)	1.52 (1.28–1.80)	<0.001	1.48 (1.20–1.82)	<0.001
Capsular invasion (Yes vs. No)	1.18 (1.02–1.37)	0.03	1.20 (0.99–1.45)	0.06
Satellite nodules (Yes vs. No)	1.30 (1.05–1.60)	0.02	1.41 (1.10–1.81)	0.01
Poor tumor differentiation (Low vs. Intermediate and high)	1.04 (0.90–1.20)	0.63	1.26 (1.04–1.52)	0.02
HBV‐DNA (>10^3^ vs. ≤10^3^ IU/ml)	1.03 (0.89–1.20)	0.69	1.28 (1.04–1.58)	0.02
AFP (>400 vs. ≤400 ng/L)	1.16 (0.99–1.35)	0.053	1.46 (1.21–1.77)	<0.001
TB (>17.1 vs. ≤17.1 μmol/L)	1.15 (0.98–1.35)	0.09		
ALB (≤40 vs. >40 g/L)	1.22 (1.04–1.43)	0.02	1.40 (1.15–1.70)	0.001
ALT (>80 vs. ≤80 U/L)	0.87 (0.68–1.13)	0.29	0.97 (0.72–1.31)	0.85
AST (>80 vs. ≤80 U/L)	1.48 (1.15–1.90)	0.002	1.34 (1.00–1.78)	0.048

Abbreviations: AFP, alpha‐fetoprotein; ALB, albumin; ALT, alanine aminotransferase; AST, aspartate aminotransferase; BCLC; Barcelona Clinic Liver Cancer staging system; CI, confidence interval; HR, hazard ratio; OS, overall survival; RFS, recurrence‐free survival; MV, multivariate; MVI, microvascular invasion; NsA, nucleoside analog; NtA, nucleotide analog; TB, total bilirubin.

* Variables found significant at *p* < 0.1 in univariable analyses were entered into multivariable Cox regression analyses.

### 3.3 Difference in OS based on NsA or NtA therapy

Among the 1303 patients, 460 (35.3%) patients died during the follow‐up period. OS in NtA group was significantly longer than in NsA group (HR, 0.38; 95% CI, 0.27–0.54; *p* < 0.001; Figure 2B). The OS rates at 1, 3, and 5 years were 15.9%, 33.0%, and 41.2%, respectively (Figure 2B).

For the 594 PSM patients, 119 (30%) patients in the NsA group died, and 34 (17.1%) patients in the NtA group died. The NtA group had significantly better OS than the NsA group (HR, 0.52; 95% CI, 0.36–0.76; *p* = 0.001; Figure 3B), and the 1‐, 3‐, and 5‐year OS rates were 85.4%, 72.0%, and 64.2%, respectively, with NsA therapy and 93.4%, 83.5%, and 80.0%, respectively, with NtA therapy (*p* = 0.001, Figure 3B).

Multivariable Cox regression analyses including 1303 patients revealed that NtA treatment was independent of other predictive factors, and the risk of death was significantly lower than NsA treatment (HR, 0.41; 95% CI, 0.29–0.59; *p* < 0.001; Table 2). Independent risk factors for death included later stage of BCLC (HR, 2.06; 95% CI, 1.61–2.62; *p* < 0.001), larger tumor size (HR, 1.87; 95% CI, 1.51–2.33; *p* < 0.001), cirrhosis (HR, 1.70; 95% CI, 1.22–2.37; *p* = 0.002), MVI (HR, 1.48; 95% CI, 1.20–1.82; *p* < 0.001), presence of satellite nodules (HR, 1.41; 95% CI, 1.10–1.81; *p* = 0.01), poor tumor differentiation (HR, 1.26; 95% CI, 1.04–1.52; *p* = 0.02), higher preoperative HBV‐DNA load (HR, 1.28; 95% CI, 1.04–1.58; *p* = 0.02), higher preoperative AFP level (HR, 1.46; 95% CI, 1.21–1.77; *p* < 0.001), lower preoperative ALB level (HR, 1.40; 95% CI, 1.15–1.70; *p* = 0.001), and higher preoperative AST level (HR, 1.34; 95% CI, 1.00–1.78; *p* = 0.048; Table 2).

### 3.4 Subgroup analysis of TDF versus ETV

**FIGURE 4:**
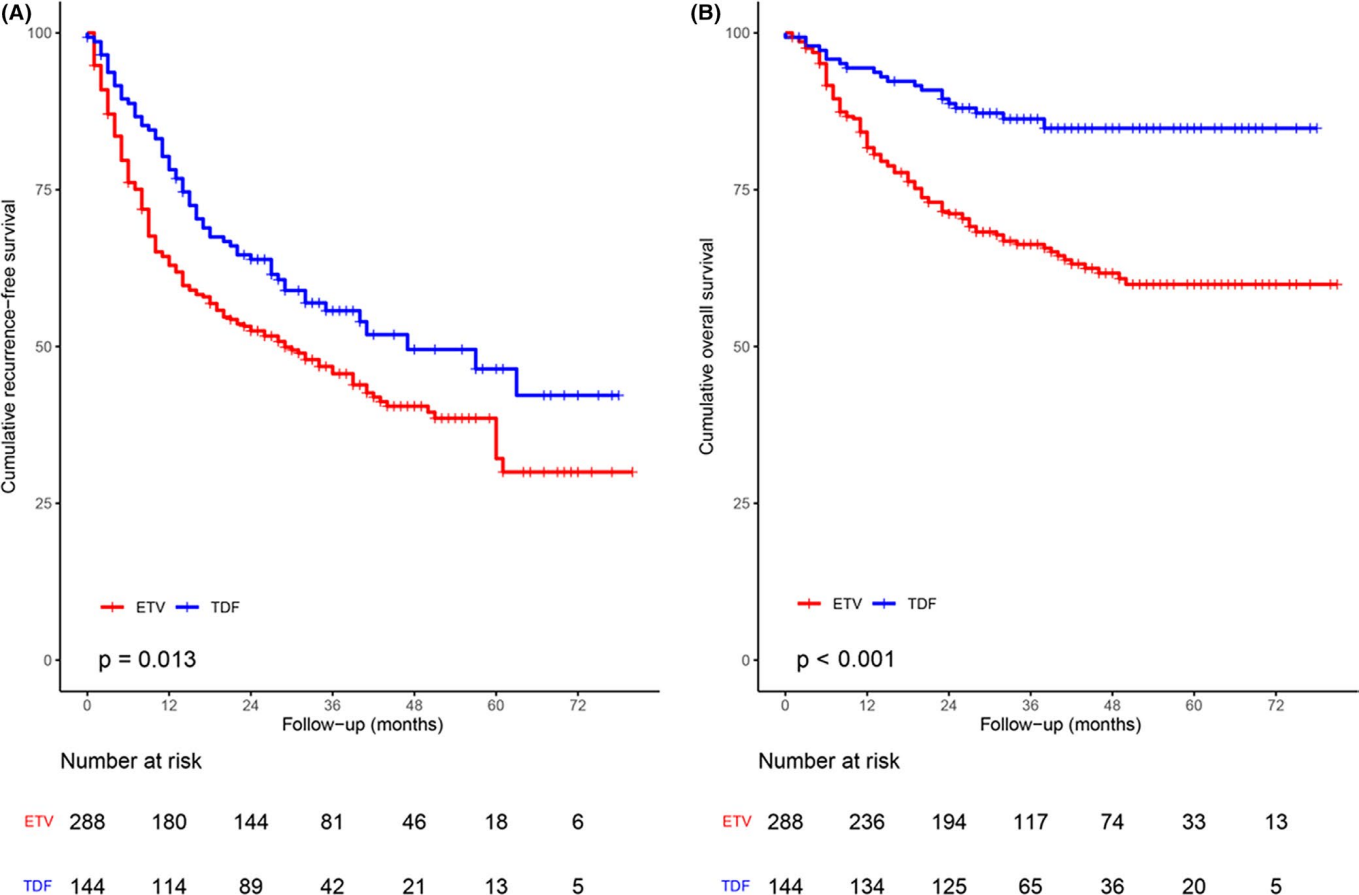
Kaplan–Meier analysis of (A) RFS and (B) OS among 432 patients receiving ETV treatment or TDF treatment. ETV, entecavir; TDF, tenofovir disoproxil fumarate

Entecavir versus TDF subgroup analysis included 432 patients (288 ETV and 144 TDF). A total of 162 (56.2%) patients in the ETV therapy group developed recurrence, and 65 (45.1%) patients in the TDF therapy group developed recurrence. The TDF group showed a significantly better RFS (HR, 0.70; 95% CI, 0.53–0.93; *p* = 0.015; Figure 4A). The 1‐, 3‐, and 5‐year recurrence rates were 37.1%, 54.3%, and 67.9%, respectively, with ETV therapy and 21.8%, 44.3%, and 53.6%, respectively, with TDF therapy (*p* = 0.013, Figure 4A).

Among these patients, 101 (35.0%) patients in the ETV group and 20 (13.8%) patients in the TDF group died. The TDF group exhibited significantly lower mortality (HR, 0.35; 95% CI, 0.22–0.57; *p* < 0.001, Figure 4B). ETV therapy resulted in 1‐, 3‐, and 5‐year OS rates of 81.7%, 66.3%, and 59.9%, respectively, and TDF therapy resulted in OS rates of 94.4%, 86.3%, and 84.8%, respectively (*p* < 0.001, Figure 4B).

The multivariable Cox regression model revealed that the TDF group showed significantly better RFS (HR, 0.64; 95% CI, 0.49–0.83; *p* = 0.001) and OS (HR, 0.32; 95% CI, 0.20–0.50; *p* < 0.001) than the ETV group, and TDF was an independent protective factor for HCC recurrence and death (Table 3). Independent risk factors for HCC recurrence included younger age (HR, 1.22; 95% CI, 1.01–1.48; *p* = 0.04), later stage of BCLC (HR, 1.32; 95% CI, 1.06–1.65, *p* = 0.02), larger tumor size (HR, 1.78; 95% CI, 1.50–2.10, *p* < 0.001), cirrhosis (HR, 1.50; 95% CI, 1.19–1.90; *p* = 0.001), MVI (HR, 1.52; 95% CI, 1.27–1.82; *p* < 0.001), capsular invasion (HR, 1.20; 95% CI, 1.02–1.40; *p* = 0.03), presence of satellite nodules (HR, 1.33; 95% CI, 1.07–1.66; *p* = 0.01), lower preoperative ALB level (HR, 1.26; 95% CI, 1.07–1.50; *p* = 0.01), and higher preoperative AST level (HR, 1.52; 95% CI, 1.17–1.97; *p* = 0.002; Table 3). Independent risk factors for death included later stages of BCLC (HR, 2.21; 95% CI, 1.71–2.85; *p* < 0.001), larger tumor size (HR, 1.85; 95% CI, 1.46–2.33; *p* < 0.001), cirrhosis (HR, 1.66; 95% CI, 1.18–2.33; *p* = 0.004), MVI (HR, 1.50; 95% CI, 1.20–1.88; *p* < 0.001), presence of satellite nodules (HR, 1.50; 95% CI, 1.16–1.95; *p* = 0.002), higher preoperative HBV‐DNA load (HR, 1.33; 95% CI, 1.07–1.66; *p* = 0.01), higher preoperative AFP level (HR, 1.46; 95% CI, 1.19–1.79; *p* < 0.001), lower preoperative ALB level (HR, 1.48; 95% CI, 1.20–1.82; *p* < 0.001), and higher preoperative AST level (HR, 1.48; 95% CI, 1.10–1.99; *p* = 0.01; Table 3).

**TABLE 3:** Multivariate analyses of RFS and OS in HCC patients receiving ETV or TDF therapy after hepatectomy

Variables	RFS		OS	
	MV HR (95% CI)	MV *p* value*	MV HR (95% CI)	MV *p* value*
Age (>60 vs. ≤60 years)	1.22 (1.01–1.48)	0.04	1.25 (0.98–1.61)	0.08
BCLC stage (B, C vs. 0, A)	1.32 (1.06–1.65)	0.02	2.21 (1.71–2.85)	<0.001
Subgroup (TDF vs. ETV)	0.64 (0.49–0.83)	0.001	0.32 (0.20–0.50)	<0.001
Tumor size (>5.0 vs. ≤5.0 cm)	1.78 (1.50–2.10)	<0.001	1.85 (1.46–2.33)	<0.001
Multiple tumors (Yes vs. no)	1.22 (0.92–1.61)	0.17	0.75 (0.53–1.06)	0.10
Cirrhosis (Yes vs. No)	1.50 (1.19–1.90)	0.001	1.66 (1.18–2.33)	0.004
MVI (Yes vs. No)	1.52 (1.27–1.82)	<0.001	1.50 (1.20–1.88)	<0.001
Capsular invasion (Yes vs. No)	1.20 (1.02–1.40)	0.03	1.21 (0.99–1.48)	0.07
Satellite nodules (Yes vs. No)	1.33 (1.07–1.66)	0.01	1.50 (1.16–1.95)	0.002
Poor tumor differentiation (Low vs. Intermediate and high)	1.02 (0.87–1.19)	0.85	1.20 (0.98–1.47)	0.09
HBV‐DNA (>10^3^ vs. ≤10^3^ IU/ml)	1.04 (0.89–1.22)	0.62	1.33 (1.07–1.66)	0.01
AFP (>400 vs. ≤400 ng/L)	1.15 (0.98–1.34)	0.10	1.46 (1.19–1.79)	<0.001
TB (>17.1 vs. ≤17.1 μmol/L)	1.13 (0.95–1.33)	0.16		
ALB (>40 vs. ≤40 g/L)	1.26 (1.07–1.50)	0.01	1.48 (1.20–1.82)	<0.001
ALT (>80 vs. ≤80 U/L)	0.83 (0.64–1.09)	0.19	1.00 (0.73–1.37)	>0.999
AST (>80 vs. ≤80 U/L)	1.52 (1.17–1.97)	0.002	1.48 (1.10–1.99)	0.01

Abbreviations: AFP, alpha‐fetoprotein; ALB, albumin; ALT, alanine aminotransferase; AST, aspartate aminotransferase; BCLC; Barcelona Clinic Liver Cancer staging system; CI, confidence interval; ETV, entecavir; HR, hazard ratio; MV, multivariate; MVI, microvascular invasion; OS, overall survival; RFS, recurrence‐free survival; TB, total bilirubin; TDF, tenofovir disoproxil fumarate.

* Variables found significant at *p* < 0.1 in univariable analyses were entered into multivariable Cox regression analyses.

## 4 DISCUSSION

In this multicenter study, we compared the clinical outcomes of 1303 patients with HBV‐related HCC treated with NtA or NsA therapy after curative resection. We found that NtA therapy was associated with decreased recurrence and increased OS compared to NsA therapy for patients undergoing R0 liver resection of HBV‐related HCC. The subgroup analysis revealed that the TDF group had higher RFS and OS rates than the ETV group. This was consistently observed in propensity score‐matched and multivariable‐adjusted analyses.

Although there has been progress in the management of HCC, the high recurrence rate of tumors remains a major problem for HCC patients undergoing curative resection. There are no internationally recognized effective adjuvant therapies to prevent the postoperative recurrence of HCC. Well‐known risk factors for HCC recurrence include advanced BCLC stage, multiple tumors, satellite lesions, large tumors, microvascular invasion, and high HBV load.^8^, ^17^ For patients with high‐risk factors for recurrence, clinical intervention should be actively pursued because these tumor characteristics are not easily improved. In addition to tumor characteristics, inflammation plays a key role in tumorigenesis, and the inflammatory microenvironment is an important part of tumorigenesis.^18^ Many carcinogenic microbial infections, such as HBV, are be associated with some forms of chronic inflammation.^19^ Therefore, host hepatitis virus load is a correctable risk factor for HCC recurrence after therapeutic resection.^8^ Previous studies showed that high levels of serum HBV‐DNA and HBsAg seropositivity were associated with the development and recurrence of HCC.^8^, ^17^ Active antiviral therapy with NUCs is an effective treatment for the prevention of HCC recurrence after curative resection.^11^, ^12^, ^20^

It has been consistently reported that oral treatment with NUCs reduces the risk of postoperative recurrence of HBV‐related HCC.^10^, ^11^, ^20^ The primary mechanism of NUC therapy is inhibition of the activity of HBV polymerase, which halts HBV replication^21^ and inhibits the direct and indirect carcinogenic mechanisms of HBV.^22^ Antiviral therapy prevents HBV reactivation, inhibits hepatitis activity, and reduces the inflammation of liver tissues, which leads to the regression of liver fibrosis and cirrhosis.^23^ Many recent studies have explored the differential effects of TDF and ETV on risk of HCC in patients with chronic HBV infection. Two meta‐analyses and two cohort studies reported that TDF treatment was associated with a lower risk of HCC than ETV treatment.^24^, ^25^, ^26^, ^27^ These studies found that the virological response rate in the early period was higher in patients treated with TDF than in those treated with ETV. This suggests that TDF may have advantages over ETV in terms of the prevention of HCC. Recently, one study showed no significant difference in the rates of HCC recurrence and death between the ETV and TDF treatment groups,^14^ and two other studies showed that the rates of recurrence and death in the TDF group were significantly lower than those in the ETV group.^13^, ^28^ None of the studies showed that ETV was better than TDF.

The potential mechanism is not clear. However, a recent study by Murata et al. showed that NtA induced the expression of interferon‐λ3 (IFN‐λ3) and inhibited the production of HBsAg.^29^ IFN‐λ exhibited effective antitumor activity in a mouse model of cancer.^30^, ^31^, ^32^ IFN‐λ3 directly inhibits tumor growth via the induction of apoptosis and/or cell cycle arrest and enhances host immunity by modulating innate and adaptive immune responses.^33^ In addition, another study by Murata et al. revealed that only NtA therapy (adefovir dipivoxil [ADV] and TDF) has an additional pharmacological effect in modulating lipopolysaccharide‐mediated cytokine production, which was expected to have a favorable immune response to the elimination of HBV.^34^ Our result is in line with the research by Choi et al.^13^ However, included NtA and NsA therapy, and they included only TDF and ETV therapy. At present, some low‐income countries/regions may have problems concerning access to TDF or the high price of TDF. Our study suggests that other NtAs, such as ADV, seem to have the same ability to reduce the risk of HCC recurrence after curative resection. For these cases, we think they can use ADV instead.

Some previous studies suggested that the serum HBV‐DNA level or HBsAg seropositivity was an independent risk factor for HCC recurrence and death.^8^, ^17^ Conversely, some studies found that serum HBV‐DNA or HBsAg was not associated with prognosis in HCC.^12^, ^35^ Our study found that a higher preoperative serum HBV‐DNA level was not an independent risk factor for HCC recurrence, but it was an independent risk factor for death. One possible reason is that all patients were treated with antiviral agents, and the relative risk of viral status was low and was overshadowed by stronger risk factors.

Our study has several limitations. First, this study was a retrospective study. Although we attempted to compensate for potential bias by propensity matching variables associated with treatment outcomes, selection bias remained a possibility. Second, in the subgroup analysis, the TDF group was comprised of much fewer patients than the ETV group because TDF was not approved for use in China until after 2016. Due to limited experience and possible renal injury, physicians might recommend TDF more strictly and carefully, leading to patient selection bias. Third, our study explored the impact of two classes of antiviral drugs on the prognosis of HBV‐related HCC, and the efficacy of antiviral agents in the same class of drugs may differ.

In conclusion, NtA, especially TDF, is a highly potent NUC that effectively reduces HCC recurrence and prolongs postoperative survival. Our study suggests that if patient circumstances permit, NtA therapy, especially TDF, should be preferred in patients with HBV‐related HCC after curative resection. Our findings may have considerable clinical significance for the prevention of HCC recurrence in patients.

## CONFLICT OF INTEREST

The authors have no conflict of interest to disclose.

## Data Availability

The data that support the findings of this study are available from the corresponding author upon reasonable request.
